# Comparative performance of agricultural productivity in 44 SSA countries for a period of 59 years (1961–2019): A Malmquist productivity index approach

**DOI:** 10.1371/journal.pone.0284461

**Published:** 2023-07-24

**Authors:** Yagbasuah Maada Baion, Wang Yunxia, Edward Hingha Foday, Cao Jianmin, Liu Chuanfu, Opoku Agyeman

**Affiliations:** 1 College of Economics and Management, Jilin Agricultural University, Changchun, China; 2 Faculty of Development Agriculture and Natural Resources Management, Eastern Technical University of Sierra Leone, Kenema City, Sierra Leone; 3 School of Environmental Science and Engineering, Chang’an University, Xi’an, Shaanxi Province, P.R China; Loughborough University, UNITED KINGDOM

## Abstract

We investigate the extent and nature of productivity growth in Sub-Saharan African countries using non-parametric frontier techniques. In this work, we examine agricultural total factor productivity (TFP) change in 44 countries in Sub-Saharan Africa (SSA), covering a period of fifty-nine (59) years (1961–2019). We make use of data drawn from the Food and Agriculture Organization of the United Nations. Due to the non-availability of reliable input price data, the study uses data envelopment analysis (DEA) to derive Malmquist productivity indices. The results demonstrate a decline in agricultural TFP growth in the region from 1961 to 2019. This notwithstanding, a general average growth was seen in some of the conventional inputs measured (land, labour, capital, and fertilizer) which are the main drivers of growth in the region. This finding implies that the increase in agricultural output over the past five decades in SSA is mainly due to an increased use of conventional inputs over time, including land, and not due to an increase in the ratio of output over inputs. As a generally acceptable and preferred indicator of technical and efficiency changes, we use TFP to calculate the Malmquist productivity index (MPI).

## 1.0 Introduction

Periodic (though in most developing countries, largely regular) hikes in food prices over the last five decades have caused actions and reactions in the global food supply chain with some dire effects, hence setting a stage for diverse disparities in the levels of demand for and supply of foodstuffs and other agricultural products across the globe. These disparities coupled with growth in human population, have increased concerns about the level of global productivity, as demand for agricultural products seems to fast outpace global supply. Worse affected are the developing regions of the world, where a vast majority of poor people lives in rural communities and largely depends on agriculture for their livelihood. As such, accelerating agricultural productivity could be a core strategy for all-round development in these regions [1].

In the Sub-Saharan Africa region for example, such factors as faltering economic growth or stagnation and decline, Steve Wiggins [2], political instabilities, or such climatic factors as famine, ecological and environmental degradation, have often resulted in failed food systems which have ultimately threatened efforts at combating food insecurity in the region and the world at large. Arguably, achieving, nurturing and accelerating agricultural productivity growth in SSA is invaluably essential for the overall development of the region. Unfortunately however, agricultural productivity in the SSA region currently falls behind other regions of the world [3–8]. Diverse hypotheses have been put forward by some of the development literatures in attempts to explain the many reasons for the underperformance of the region’s agricultural sector.

Fulginiti and Perrin [9], examining a group of Least Developed Countries (LDCs) from 1961–1985, did not report direct measures of productivity change. However, the results obtained from their Cobb-Douglas production measurement showed technological regression for 14 of the 18 countries. Kawagoe et al. [10], using data for 1960, 1970, and 1980 in 21 developed countries (DCs) and 22 (LDCs), estimated cross-country production functions with dummy variables for 1970 and 1980. They also reported technological regression during both decades for the LDCs, but technological progress in the DCs. Kawagoe et al. [10] reported similar results in that data set using indirect production function. Results from the work of Lau and Yotopoulos [11] also showed positive productivity during the 1960s but negative productivity for LDCs during the 1970s. Lilyan E. Fulginiti and Richard K. Perrin [12] observed changes in agricultural productivity in 18 developing countries over the period 1961–1985, with the use of a nonparametric, output-based Malmquist index and a parametric variable coefficients Cobb-Douglas production function to examine whether their estimates conform to other results from other studies that have indicated declining agricultural productivity in LDCs.

The principal aim of this study is to provide up-to-date information on agricultural total factor productivity (TFP) growth over the past five decades (1961–2019) for 44 Sub-Saharan African countries. It should be noted that the study by Wiebe et al. [13] does analyze TFP growth for 110 nations over the 1961–1997 period. His study left out most Sub-Sahara African countries. However, he does use the Cobb–Douglas production function, which introduces several restrictive assumptions, such as constant production elasticities (and hence input shares) across all countries, Hicks-neutral technical change, and the requirement that crop and livestock outputs be aggregated into a single output measure. We contend that further and consistent review and assessment of the situation is crucial for providing policy and decision makers both in governments and the private sector with sufficient and necessary information for informed judgments on policy formulations and/or shifts aimed at addressing the issues of declining agricultural productivity in the region. Therefore, the analysis in the present study uses the DEA technique to calculate the Malmquist TFP index. This method does not make any of the above assumptions. However, it is susceptible to the effects of data noise and can suffer from the problem of “unusual” shadow prices, when degrees of freedom are limited.

In this research, we compare the performance of agricultural productivity using the Malmquist Index (MI), considering its enhanced applicability approach. This paper investigates the TFP level of 44 SSA countries. Also, this research seeks to determine the agricultural productivity change among SSA countries and the causes of the change(s), if any. The remainder of this paper is organized into sections: Section 2 describes the DEA and Malmquist TFP index methods, section 3; presents the data used, while the empirical results are discussed in Section 4, and concluding comments are made in the final section.

## 2.0 Methodology

Using similar Malmquist Index methods outlined in Fare et al. [14, 15], the TFP was calculated. This method uses DEA methods to create a piece-wise linear production frontier for each year in the sample. For this work, we employ a time series of 162 categories of crop commodities, 30 animal and insect, and 8 aquaculture products as our output variables and 6 categories of inputs prices to construct a Malmquist DEA Index of TFP for a period of 59 years (1961–2019). Consequently, a summary of TFP is provided together with DEA techniques before a description of the Malmquist TFP computations.

### 2.1 Total Factor Productivity (TFP)

Similar to the one put forward by Fuglie, K.O., and N. Rada [16], we measured productivity with the use of total productivity indices, which reflect the various contributions of all the conventional factors of production (land, labour, capital, and materials). In other words, TFP measures the rate of growth and/or decline in productivity over time. More outputs in relation to the quantity of a given set of inputs over time is indicative of a TFP growth and the opposite is correct. TFP measurements may be a complex approach to the challenges of agricultural productivity, but it is a much more inclusive and detailed form of measurement than partial measurements. TFP methods have gained much popularity and are being widely used for growth analysis. This is largely due to the heightened interests that policymakers, decision-makers, politicians, economists, and scientists have shown in understanding both agricultural productivity growth patterns and their possible sources. Estimation of TFP growth for most countries, especially developing ones, can be tasking owing to the scarcity (or the lack, in some cases) of readily available and adequately representative data, on input and output quantities for carrying out such estimations [17].

Total factor productivity is defined as the ratio of output and inputs. Therefore, growth in TFP is the residual share of output growth after accounting for percentage changes in land, labour, and other conventional factors of production [18], let *Y* represent total output and *X* represent total inputs, TFP can thus be given as:

$$TFP=\frac{Y}{X}$$ (1)

Given the difficulties surrounding the establishment of significant definition for actual output or inputs, index number theory is often used to provide a significant meaning for output and inputs changes between any two given periods of time [19]. This is often done by comparing the rate of change in total output with that of total inputs. Eq (1) can therefore be rewritten as:

$$\frac{dIn(TFP)}{dt}=\frac{dIn(Y)}{dt}-\frac{dIn(X)}{dt}$$ (2)

meaning that the rate of change in TFP is the difference between the rate of changes in both the aggregated output and inputs. Since agricultural productivity involves a combination of multi-output and multi-inputs, *Y and X* are hence the vector of the process. Using a constant returns to scale (CRS) production function, producers seek to maximize outputs, therefore, the output elasticity in relation to a given set of inputs, equals the cost share of the inputs, hence setting up the market for a competitive equilibrium in the long run, making revenue equal to total cost. As such, Eq (2) can be rewritten as:

$$In(\frac{{TFP}_{t}}{{TFP}_{t-1}})=\sum R_{i}\;In(\frac{Y_{i,t}}{Y_{i,t-1}})-\sum S_{j}\;In(\frac{X_{j,t}}{X_{j,t-1}})$$ (3)

where *R*_*i*_ and *S*_*j*_ are the revenue share of the *i*th output and the cost share of the *j*th input, respectively. Total output is thus the sum of the growth rate of each output commodity as weighted by its revenue share and total input is similarly the sum of the growth rate of each input, weighted by its cost share. Therefore, TFP is the value-share-weighted difference between total output growth and that of total input growth.

Growth in TFP can be assessed using two key parameters; first, TFP can grow when the firm or industry is able to use its existing inputs or technology to improve its outputs over time and this is known as efficiency change (EFFCH or EC). EC also known as Catch Up, is productivity dynamics due to managerial performance, decisions, and skills. It is managerial efficiency over time, and it is also known as the movement of the firm towards the production possibility frontier (PPF) and usually shows the firm’s ability to improve to a point of getting closer to or hitting the PPF. EC is thus expressed as:

$$EC(a,b)=\frac{\emptyset^{bb}}{\emptyset^{aa}}\leq or>1$$ (4)

where ∅^*bb*^ is the score of the second year divided by ∅^*aa*^ the score of the first year. Where the DMU is closer to the frontier in the second period (period ∅^*bb*^) than it was in the first period (period ∅^*aa*^), then the catching up index = 1, in other words, the said DMU is catching up with the frontier (improving). Also, if the index is >1, that shows that the DMU has improved. But if the index is <1, this is an indication of a decline relative to the frontier or the best performers. Therefore, EC is the ratio of the own period efficiency for the second year divided by the own period efficiency for the first year.

Assuming:

$$A^{aa}=0.3$$ (5)

$$A^{bb}=0.4$$ (6)

therefore,

$$EC=\frac{0.4}{0.3}$$ (7)

EC=1.33 (8)

where:

*A*^*aa*^ = 0.3 is the own period efficiency score for the first year and

*A*^*bb*^ = 0.4 is the own period efficiency score for the second year.

This shows that DMU *A* has improved its efficiency by 33% in the second year, relative to the first year. Note that even though DMU *A* is inefficient in both period one and two, EC estimation helps us realize that DMU *A* has actually improved and such improvement is attributable to the efficient management of the firm.

Second, TFP grows when there is an adoption of innovative skills and ideas such as improved designs, research and development, electronics, mechanization, process or product development and the like, in the production circle. This parameter, also known as technical or technological change (TECHCH) or frontier shift (FS) is a shift in the PPF and it is a production dynamic attributable to technological dynamics within the industry or firm. FS talks about those visible and invisible technological dynamics outside the control of the management that influence the behavior of the firm. It is therefore the difference between DMU *A* being projected onto frontier *a* and onto frontier *b* (frontier is a set standard against which a DMU or organization’s performance is benchmarked or measured at a given time). TECHCH is expressed as:

TC(a,b)=∅aa∅ab∅ba∅bb12≤or>1 (9)

TC=babb×aaab12≤or>1 (10)

where:

TC is the square root of the four indices

(a, b) represent two observations or DMUs

∅^*aa*^ is the own period ∅ of a DMU in time period ‘a’ relative to frontier ‘a’

∅^*ab*^ is the own period ∅ of a DMU in time period ‘a’ relative to frontier ‘b’

∅^*ba*^ is the own period ∅ of a DMU in time period ‘b’ relative to frontier ‘a’

∅^*bb*^ is the own period ∅ of a DMU in time period ‘b’ relative to frontier ‘b’

### 2.2 Data Envelopment Analysis (DEA)

Data Envelopment Analysis (DEA) (a non-parametric approach) and Stochastic Frontier Analysis (SFA) (a parametric approach) models are the basic performance evaluating tools. [20, 21]. Here, our focus is on the DEA method as the model for this work. Our choice of the DEA for this project, as opposed to the SFA, could be deduced from the many advantages the former exhibits over the latter. DEA is a nonparametric (no parameters, boundless,) benchmarking, linear programming, frontier optimization technique used to model a relationship between (physical) inputs and outputs which defines efficiency levels.

**Fig 1:**
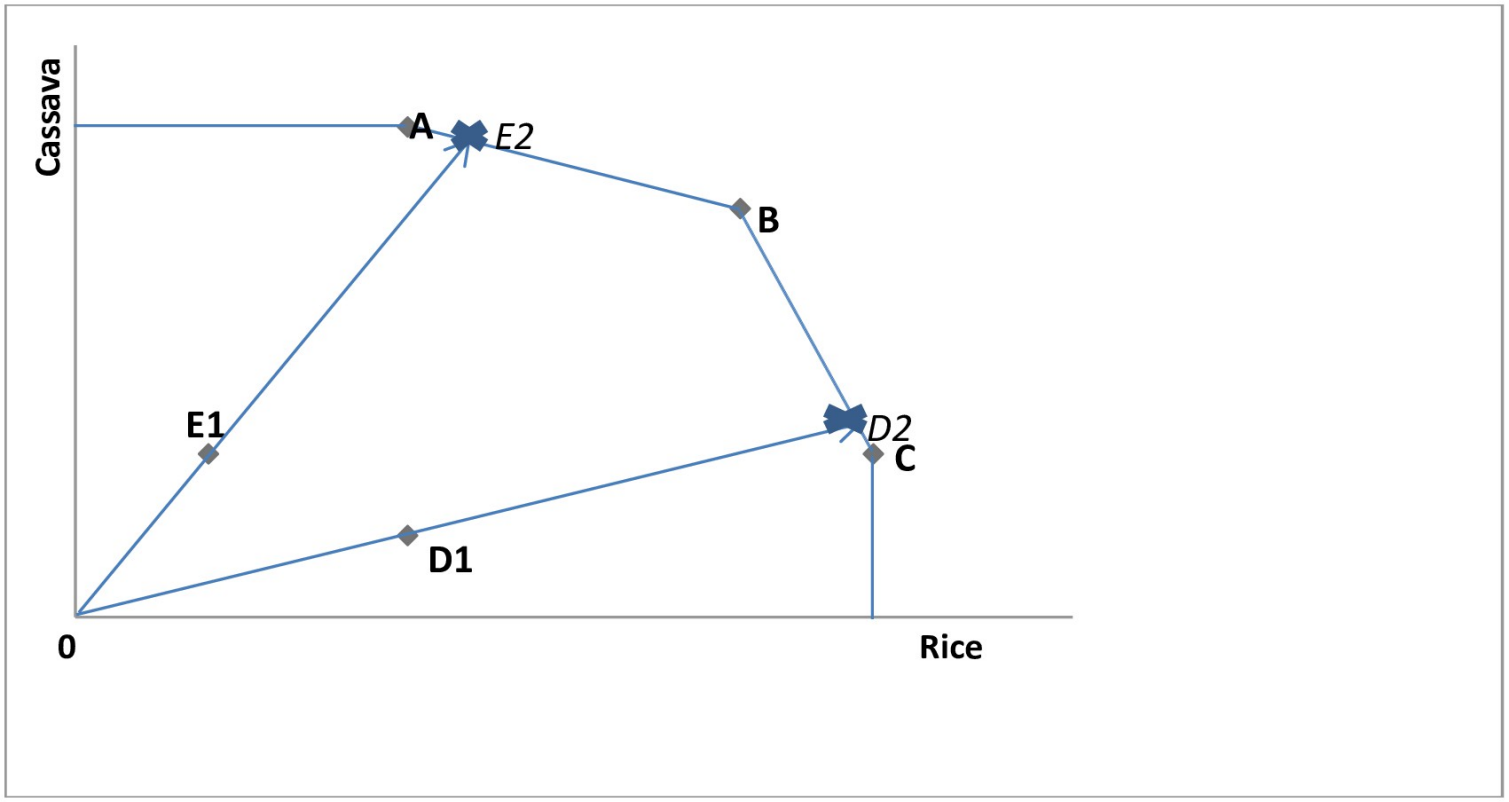
Output-oriented DEA.

In a DEA analysis, one of two basic assumptions is often made; a Constant Returns to Scale (CRS) or a Variable Returns to Scale (VRS) assumption, resulting in two different frontiers depending on which of the assumptions is adopted. However, the efficiency scales of decision-making units (DMUs) (DMUs are the units under observation, e.g. Schools, banks, cities or countries) remain unchanged in both input and output orientations when a CRS is assumed, but there are variations in efficiency scales (between input and output orientations) when a VRS is assumed. In this work, a CRS technology is assumed. In their work, Grifell-Tatje and Lovell [22, 23] use a simple one-input, one-output example to demonstrate that a Malmquist TFP index may not accurately measure TFP changes when a VRS is assumed. As such, it is imperative that the CRS technology is assumed for such estimation; otherwise, an inaccurate result may be imminent and may not properly reflect the TFP gains or losses resulting from scale effects. It is important to note that in a DEA, maximizing outputs whilst units of inputs are held constant determines the efficiency of the best practice DMU. Technical efficiency is the ability of a firm (or country as it is in our case) to reduce its input while holding the output constant, or augment its output whilst holding its input constant, and it is often measured in relation to the distance from the origin to the frontier divided by the distance from the origin to the DMU, over time, as illustrated in Fig 1. This permits the construction of a Malmquist Index of TFP, from the efficiency measures without the use of a price domain, thus enhancing efficient inter-country comparison over time.

DEA is widely associated with two basic models [24]; Charnes A, Coopers W, and Rhodes (CCR) model [25] and Banker, R. D., Charnes, A., & Cooper, W. W (BCC) model [26]. The BCC model is widely accepted as an extensive work on the original CCR model. For the purpose of this work, we explore the CCR model as propounded by Charnes et al [25]. Further, we adopt the output orientation in line with the objective of our study of estimating the agricultural TFP and performance levels of countries in the SSA region.

The DEA model could better be illustrated here; supposed you are served a set of time series data on five (*N*) DMUs producing two outputs (rice and cassava), using an output-oriented DEA, the possible linear programming problem (LPP) that is solved for the *i* − *th* DMU is as follows. DMUs A, B and C are said to be technically efficient because they all operate on the production frontier, whereas DMUs E and D are dominated by the frontier and are thus (in relation to A, B and C) inefficient. Each of these efficient DMUs (A, B & C) has an efficiency score that is equal to unity or one and are the benchmarks or peers [27] to the inefficient DMUs.

We titled the two X’s on the frontier E2 and D2 respectively to give a better understanding to our readers of the aspired points of efficiency for both DMU E and D. In order therefore for DMU *E*, for example, to attain efficiency, it will need to augment its output level by moving from its current position on the graph (Point *E*1) towards the production frontier and hit the region between *DMUs A and B* whilst at the same time holding its input constant. *DMU E*’ *s* efficiency score is thus equal:

$$\phi_{E}=\frac{distance\;from\;the\;origin\;to\;the\;frontier}{distance\;from\;the\;origin\;to\;the\;DMU}\geq 1$$

Or for shorthand:

$$\phi_{E}=\frac{0E1}{0E2}\geq 1$$ (11)

DMU *E* could therefore obtain an output-oriented technical efficiency of approximately 3 or 30%, leaving it 70% (approximately) short of the efficiency scores displayed by its peers (*DMUs A* & *B*). Similarly, *DMU D* can achieve efficiency by moving from its current position (*Point D*1) towards the production frontier, hitting a point somewhere between *DMUs B* & *C* and its efficiency score can be expressed as:

$$\phi_{D}=\frac{distance\;from\;the\;origin\;to\;the\;frontier}{distance\;from\;the\;origin\;to\;the\;DMU}\geq 1$$

Or for shorthand:

$$\phi_{D}=\frac{0D2}{0D1}\geq 1$$ (12)

DMU D could have an output-oriented efficiency score of approximately, 4 or 40%, suggesting that the DMU is 60% short of the efficiency scores of its peers (*DMUs B* & *C*).

### 2.3 The Malmquist index

The Malmquist productivity index, centered on the function of production maximization, is used for measuring the dynamics of productivity over a period of time [28]. It is often constructed by taking a weighted average of diverse series of data. In contrast to the conventional function of production and other indices, the Malmquist index makes a clear distinction of two main sources of productivity increase or decline–changes in technical efficiency (catch-up) and changes in production technology (frontier shift effect). These are mostly used in studies estimating agricultural TFP dynamics at farm, national, regional and global levels.

In recent years, the Malmquist index has become the standard approach to productivity measurement within the non-parametric literature [29]. The Malmquist Productivity Index (MPI) is a bilateral index (used to compare two production periods at a time) which was named after Professor Sten Malmquist. It is used in assessing the productivity changes in organizations or countries over time. MPI can also be used to compare productivity changes within two heterogeneous economies over time. In general, MPI is concerned with efficiency studies over time. This index, rather than just observe in brief the performance of organizations at one specific time, goes as far as reflecting the performance (change) of organizations across different time periods. Introduced by Caves et al. [19], the MPI is used as ratios of distance functions; a reciprocal of technical efficiency [30]. This makes possible the description of multiple inputs and multiple outputs production technology without recourse to specification of behavioral intent that has to do with for example, the minimization of cost or augmentation of revenue. A distance function could either be input or output (distance function), whereas both could be defined and used in similar manner, our preoccupation in this work is with the output distance function in line with our objective of output augmentation whilst holding input constant (output orientation). An output distance function looks at the utmost proportionate expansion of the output vector of a DMU, given an input vector.

Assume a production technology with an output set of *P*(*x*), where the input vector *x* is used to produce the output vector *y*. That is,

$$P\left(x\right)=\left\{y:x\;can\;produce\;y\right\}.$$ (13)

Note, that the axioms itemized in Coelli et al [31] are assumed to satiates the technology.

Hence, the output distance function is defined on the output set, *P*(*x*) as follows:

$$d_{0}\left(x,y\right)=min\left\{\delta :\left(\frac{y}{\delta }\right)\epsilon P\left(x\right)\right\}$$ (14)

The distance function *d*_0_(*x*, *y*), will assume a value ≤ unity where the output vector *y* is a component of the feasible production set, *P*(*x*). It will assume similar value, if *y* is located on the outer boundary of the feasible production set, this is consistent with the convexity axiom. The distance function *d*_0_(*x*, *y*), will however assume a value ˃ unity if *y* is located outside the feasible production set.

The Malmquist productivity Index (MPI) is based on distance functions; a reciprocal of technical efficiency. The Malmquist TFP Index is used to measure the TFP change existing between two sources of data by calculating the ratio of the distance of each of the data points in relation to a shared technology. As postulated by Fare et al [32], the Malmquist (output-orientated) TFP change index between period *s* (which is the base period) and period *t* (the own period) is given as follows;

$$m_{0}\left(y_{s},x_{s},y_{t}x_{t}\right)={\left[\frac{d_{0}^{s}(y_{t},x_{t})}{d_{0}^{s}\left(y_{s},x_{s}\right)}\times \frac{d_{0}^{t}(y_{t},x_{t})}{d_{0}^{t}\left(y_{s},x_{s}\right)}\right]}^{1/2},$$ (15)

where *m*_0_(*y*_*s*_, *x*_*s*_, *y*_*t*_ *x*_*t*_) represents the two different time period efficiencies evaluated: the cross or base period (with the *s* superscripts) and own period (with the *t* superscripts) for both the output and input sets. The MPI within the square brackets is the geometric mean of two efficiency change (EC) ratios, where one of the EC is measured relative to frontier *s* and the other is measured relative to frontier *t*. A value of MPI or *m*_0_ greater than one (˃1), indicates TFP growth and a value of MPI or *m*_0_ less than one (<1), indicates decline in TFP [33–35].

This equation can be rewritten as thus:

$$m_{0}\left(y_{s},x_{s},y_{t},x_{t}\right)=\frac{d_{0}^{t}\left(y_{t},x_{t}\right)}{d_{0}^{s}\left(y_{s},x_{s}\right)}{\left[\frac{d_{0}^{s}\left(y_{t},x_{t}\right)}{d_{0}^{t}\left(y_{t},x_{t}\right)}\times \frac{d_{0}^{s}\left(y_{s},x_{s}\right)}{d_{0}^{t}\left(y_{s},x_{s}\right)}\right]}^{1/2},$$ (16)

where the ratio outside the square brackets estimates the efficiency change in the output-oriented measure of the technical efficiency of Farrell, [36], between periods *s* and *t*. In other words, the efficiency change index is equivalent to the ratio of the Farrell technical efficiency in period *t* to the Farrell technical efficiency in period *s*. The outstanding portion of the index in Eq 16 is a measure of technical change. It is the geometric mean of the shift in technology between the two periods, being estimated at both *x*_*t*_ and *x*_*s*_.

With a suitable panel data at our disposal, we can calculate the required distance measures for the Malmquist TFP index by means of DEA-like linear programs, following the leading of Fare et al [32]. For the *i*th DMU, we must conduct calculation of four distance functions to measure the TFP change between periods, *s* and *t*. That is, four different linear programming problems (LPP) as required by the Malmquist productivity index are to be solved. The required linear programming problems are solved as thus:

$$\begin{array}{l} {\left[d_{0}^{t}\left(y_{t},x_{t}\right)\right]}^{-1}=max_{\phi ,\lambda }\phi ,\\ st-\phi y_{it}+Y_{t}\lambda \geq 0,\\ x_{it}-X_{t}\lambda \geq 0,\\ \lambda \geq 0 \end{array}$$ (17)

$$\begin{array}{l} {\left[d_{0}^{s}\left(y_{s},x_{s}\right)\right]}^{-1}=max_{\phi ,\lambda }\phi ,\\ st-\phi y_{is}+Y_{s}\lambda \geq 0,\\ x_{is}-X_{s}\lambda \geq 0,\\ \lambda \geq 0, \end{array}$$ (18)

$$\begin{array}{l} {\left[d_{0}^{t}\left(y_{s},x_{s}\right)\right]}^{-1}=max_{\phi ,\lambda }\phi ,\\ st-\phi y_{is}+Y_{t}\lambda \geq 0,\\ x_{is}-X_{t}\lambda \geq 0,\\ \lambda \geq 0, \end{array}$$ (19)

$$\begin{array}{l} {\left[d_{0}^{s}\left(y_{t},x_{t}\right)\right]}^{-1}=max_{\phi ,\lambda }\phi ,\\ st-\phi y_{it}+Y_{s}\lambda \geq 0,\\ x_{it}-X_{s}\lambda \geq 0,\\ \lambda \geq 0. \end{array}$$ (20)

In the LPPs of 17 and 18, the production points are calculated relative to the own period technologies and in LPPs 19 and 20, the production points are compared with the technologies from the base period and the *ϕ* parameter needs not be greater than or equal to one (≥1), as it must be in the case of standard output-orientated technical efficiencies. The data point could lie above the production frontier. This could possibly occur in LP 20 where a comparison of a production point from period *t* is made to technology in the base period, *s*. A value of psi less than unity (*ϕ*<1) is returned where there is a technical progress. This could also be possible in Eq 19 when there is technical regression.

## 3.0 Data

The research is based on data gathered from the FAOSTAT system of the FAO Statistic Division’s FAOSTAT Agriculture. All essential data may be accessed and downloaded from the FAO’s website [37]. The following are some of the characteristics of the data series used:

1. FAOSTAT: here, we were able to access information on the Gross Value of agricultural output (crops, livestock, and aquaculture) and input (land, labor, capital, livestock, machinery, and fertilizer) $1000 at constant 2015 prices in the countries and territories used in this work.
2. The Economic Research Service of the United States Department of Agriculture (USDA): Data on machinery was largely obtained from here.
3. FAO-FISHSTAT: we obtained the data on the gross value of 8 aquaculture products used in the study, estimated at $1000 at the constant 2015 global average farm-gate price, from FAO-FISHSTAT.
4. International Fertilizer Association (IFA): information on fertilizer for agricultural purposes was largely obtained here. For small countries/territories IFA only reports regional totals; fertilizer use is apportioned among these entities as a share of the total crop area harvested.

Access was granted to us to download all the necessary data from the websites of the above organizations.

### 3.1 Country coverage

The study covers 44 countries in the SSA region largely targeting the West, East, South, and Central regions of Africa. These countries we believe are the major agriculture producers on the African continent and account for over 80% of the continent’s population, hence constituting a fair representation of the continent.

We initially intended to carry out a comprehensive study on all the countries in Africa, but our study was refocused on the SSA region, and the number of countries reduced to 44 and those countries not included in this study were not included largely due to either one of the factors listed below, or an inconvenient blend of both:

- Inadequate data: some of the countries are dropped largely for lack of adequate and necessary data as required for this project.
- Inadequate arable land, hence the low interest in the agricultural sector: some of the countries are dropped for their evident lack of interest in the agricultural sector based on their levels of investment in the sector. This we believe is largely due to the fact that these countries are grossly inadequate in terms of arable land.

### 3.2 Time series

This research covers a period of 59 years (1961–2019), which we believe is in itself a novelty as no known work on the SSA region’s agricultural TFP changes with such a wider time-series coverage is available at the moment.

### 3.3 Output variables

The initial design of this work was such that three output variables: crop output, animal output, and aquaculture output could be separately measured using the DEAP software. This, however, was altered due to difficulties we encountered in running the data error-free, particularly figures recorded for aquaculture output. These sets of data constituted lots of zero values which we believe was responsible for the erroneous results obtained initially. We discovered that most of the countries under review either do not have information on their aquaculture output or were not at all involved in aquaculture farming (especially between 1961 and the mid-1980s), hence such information was nonexistent and could not be recorded. For this study, these three output variables are aggregated into a unitary value to create a single unit of output value.

**Crop output:** consists of the harvested quantity of 162 different crops, expressed in $1000 constant 2015 global average farm-gate price [38].
**Livestock output:** makes up the gross value of 30 animal products, at $1000 constant 2015 global average farm-gate price.
**Aquaculture output:** Consists of the gross value of 8 aquaculture products, at $1000 constant 2015 global average farm-gate price.

The aggregation of these 200 outputs is carried out using the global average prices from 2014–2016 in Purchasing Power Parity (PPP) dollars, to obtain the gross value of SSA agricultural products using the Geary-Khamis methodology as expressed by Rao. 1996 [39]. Eight distinct categories of products from aquaculture as estimated by the FAO-FISHSTAT are valued in US dollars which are divided by the total product value of all 44 countries to give the average unit value of each of the product groups. These values are then averaged using the 2014–2016 time periods which are set at 2015 constant dollar prices which correspond with the Paasche quantity index. Note that the output commodities consist of both food and non-food items and do not include hay, fodder, and most ornamental crops.

Tables A1 and A2 in S1 Appendix highlight the output mix for all the 44 countries involved in the research for the years 1961 and 2019 respectively. They give a breakdown of all three output variables used in the study; Columns 2, 3, and 4 of each of the tables give individual country output values for the crop, animal, and aquaculture, respectively. These output values indicate the agriculture production strength/focus of each of the countries, with some countries tending to focus largely on crop production, others on animal, and some a mix of both, but with no indication of any single country giving aquaculture a preferential status over either crop or animal production. We also discovered that in 1961, only two, out of the 44 countries had any record data for the aquaculture sector, but by 2019, only 6 out of the 44 countries were without record data for that sector. This we believe is a leap forward in that sector in the region.

In Table A3 in S1 Appendix, we do a compressive analysis of the trends of growth of all three output categories aggregated into a single output unit for the years 1961 and 2019. Columns 3, 4, and 5 show 1961 and 2019 output values and percentage changes respectively. We discovered that only seven of the 44 countries recorded a percentage change in their output of less than 100% between 1961 and 2019, with Namibia recording the least percentage change of 11%. Malawi is rated to have had the highest percentage change of 1295.5% between 1961 and 2019.

**Fig 2:**
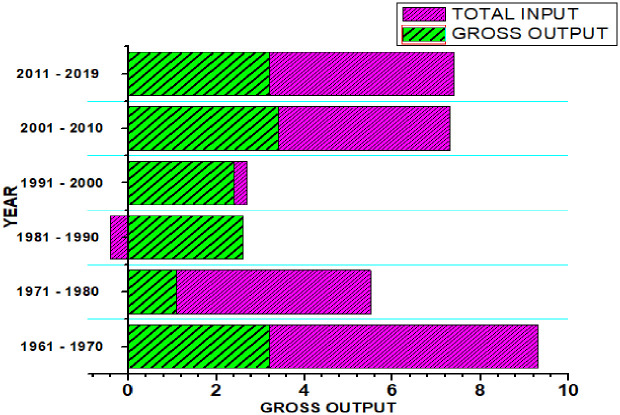
Decade-long (except for 2011–2019) output and input changes between 1961–2019.

Fig 2 gives a summary of decade-long trends (except for 2011–2019, which covers nine years) and level of changes in both the output and input variables during the study period. We noticed an overall relatively fluctuating average annual growth in the output variables during the study period with the highest average annual output growth (3.4%) recorded between 2001 and 2010 and the lowest growth (1.1%) recorded between 1971 and 1980. A steady, decline in input variables was also discovered at some points during the study period. Between 1961 and 1970, there was an average annual input growth of 6.1% which was reduced to 4.4% between 1971 and 1980. We saw a massive decline of -0.4% between 1981 and 1990, hitting its lowest ebb for the entire study period. This figure grew by 0.3% during the subsequent decade (1991–2000) and from that point on we saw a relatively steady increase in annual percentage input quantity from 3.9% between 2001 and 2010 to 4.2% between 2011 and 2019. In general, we discovered that the total average annual percentage change in input outstripped the average annual percentage change in output during the study period. This, to a larger extent, suggests that growth in total input during the study period does not correlate directly with the growth in total output.

**Gross Output:** FAO Gross Value of agricultural output from crops, livestock, and aquaculture, $1000 at constant 2015 prices, decomposed by the author into decade-long percentage change. Total Input: Aggregation of land (1000 hectares of rain fed-cropland-equivalents), labor (1000 persons (male and female) age 15years and above actively engaged in agriculture), capital, fertilizer inputs ($1000 at constant 2015 prices), livestock and machinery, as provided by FAO and decomposed into decade-long percentage changes by the author.

#### 3.3.1 Input variables

We identified six (6) input variables for this work, which are the aggregate quantity of land, labor, capital, livestock, machine, and fertilizer, as explained below:

**Land**: Land variable covers the total of agricultural land and it is measured in hectares of rain fed cropland equivalents, and this constitutes all rain-fed cropland (weight = 1.00), irrigated cropland (weight = 1.00) and permanent pasture (weight = 0.4) in each of the assessed countries.
***Rain-fed Crop Land***—Cropland in Sub-Saharan Africa as described by FAO includes areas harvested for all crops (FAO, 2019).
***Irrigated Cropland***–Land areas artificially designed and equipped for irrigation (FAO, 2019)
***Permanent Pasture Land***–Covers area in permanent pasture (FAO, 2019)
**Labor**: Labor in this work refers to the number of adults (measured in 1000 persons (male and female) age 15years and above) that are economically active or gainfully employed in the agricultural sector (modeled estimates from ILO, May 2018 update, for 1991–2020).
**Capital:** Capital is defined as the total value of a net capital stock given in a thousand dollars ($1000) at constant 2015 prices. FAO FAOSTAT Net Capital Stock (1995+) is obtained from the sum of past capital investments depreciated for wear & tear estimated using the Perpetual Inventory Method (PIM). Pre-1995 estimates were derived from records of livestock and machinery capital.
**Livestock**: Livestock accounts for the total number of livestock capital on farms in "cattle equivalents" based on the relative size and feeding requirements. Stocks of end-of-year records held on farms are obtained from FAO. Weights for each species considered in this category are obtained from Y. Hayami and V.W. Ruttan, [40] where dairy cattle are given a representative weight of 1.000. Species and their respective weights included are camels (1.100), other camelids (0.250), horses (1.000), mules (1.000), asses (0.800), dairy cattle (1.000), other cattle (0.800), goats and sheep (0.100), pigs (0.200), and poultry (10.0 per 1000 head).
**Machines**: This is the total stock of farm machinery in "40-CV tractor equivalents" (CV = metric horsepower), aggregating the number of 2-wheel tractors, 4-wheel tractors, and combine harvesters and threshers.

The types of machinery considered in this study assumed the following weights; 2 wheel tractors average 12 CV, 4-wheel tractors 40 CV, and combine-harvesters 20 CV. Data are from FAO except for 2-wheel tractors, which were compiled from (USDA ERS, 2021) [38]. Data on the number of farm machines held on farms are from FAO for 1961–2009. Post-2009 data are from (i) national agricultural censuses, (ii) accumulated commercial sales of new tractors and combines (assuming a 10-year life span), and (iii) modeled estimates of growth based on changes in per capita input and cropland/worker in agriculture.

**Fertilizer**: Metric tons of N, P2O5, and K2O fertilizer consumption. Data on N, P2O5, and K2O fertilizer consumption are from the International Fertilizer Association (IFA). For small countries/territories IFA only reports regional totals; fertilizer use is apportioned among these entities as a share of the total crop area harvested.

The above information is largely retrieved from FAOSTAT which has been publishing annual global agriculture productivity estimates since 1961, except for countries that came into being after 1961. In those countries/territories that officially came into existence after 1961, records on their agricultural productivity started from their time of independence.

## 4.0 Results and analyses

The results of our DEA calculations were acquired using DEAP version 2.1 as developed by Tim Coelli [41]. Via this computer programing, we measured the Efficiency Change (effch), Technical Change (techch), and Total Factor Productivity Change (tfpch) for each of the 44 countries for a period of 59 years. The means of the measures of efficiency change, technical change, and total factor productivity change for each of the 44 countries for the years 1962 and 2019 are presented in Tables A4 and A5 of S1 Appendix respectively. In Tables A6 and A7 in S1 Appendix, we present the Malmquist Index Summary of Annual Means and Malmquist Index Summary of Country Means (1961–2019), respectively.

Our DEA results for 1962 indicate that only 21 (less than 50%) of the 44 countries under observation experienced positive total factor productivity change largely as a result of technological progress. Rwanda, which has the highest average TFP score of 1.185 (18.5%), did so largely due to an 18.5% change in technological progress. The country with the lowest average TFP growth is Gabon, which registered an average TFP decline of 0.414 (-58.6%) mainly due to a 0.414 decline in technology. Our results show an average general annual productivity decline of 0.978 (-2.4%) for the year 1962, largely stemming from a 0.978 technological regression.

Comparatively, our DEA results for 2019, show improvement in the average TFP change with a score of 1.014 (1.4%), largely resulting from a 1.027 (27%) progress in efficiency, meaning that in 2019, countries became more efficient in the utilization of their existing inputs as opposed to the adoption and/or deployment of latest technologies. That said, our results show that of the 24 countries (over 50%) which defined the PPF in 2019, 13 (54.1%) sourced their growth largely from technological progress, suggesting that these countries possibly adopted multi-technological practices which aided their progress, but were in the actual sense inefficient. Sao Tome and Principe, with an average TFP score of 1.282 (28.2%) has the highest TFP growth for the year 2019, thus replacing Rwanda, which got the highest TFP score of 1.185 (18.5%) in 1962. A 39% efficiency change largely accounted for the TFP growth experienced by Sao Tome and Principe. This means that Sao Tome and Principe became more efficient with its existing inputs in 2019. Côte d’Ivoire returned a declined TFP score of 0.836 (-16.4%) in 2019, which made the country the least performing country for that year. Our results indicate that such decline was due to a 0.836 (-16.4%) decline in technology. However, even in the midst of average annual TPF growth in 2019, it is worth noting that 9 of the 21 countries which defined the PPF in 1962, suffered a TFP decline in 2019. However, Gambia, Mauritania, Gabon, Sao Tome and Principe, Kenya and Mauritius, all of which suffered TFP decline (with Gabon as the least performing country) in 1962, recorded positive TFP growth in 2019, three of them (Gabon, Kenya, and Mauritius) largely as a result of technological progress.

Our Malmquist index summary of annual and country means for the period under review as presented in Tables A6 and A7 of S1 Appendix respectively, suggest average annual TFP decline of 0.982 and 0.977 respectively in the SSA region. Such declines are largely attributed to a decline in technology during the reviewed period. Further, our TFP results as presented in Tables A4-A7 of S1 Appendix suggest that average annual TFP decline in the SSA region is mainly as a result of technological regression. In 1962 for instance (see Table A4 in S1 Appendix), 21 countries defined the PPF, but only 4 (19%) of them (Togo, Burkina Faso, Angola and Malawi) sourced their growth largely from progress in efficiency change and 17 (81%) of them, resulting largely from technological progress. In 2019, as illustrated in Table A5 of S1 Appendix, although the region experienced TFP growth which was largely as a result of a 1.027 (2.7%) technical efficiency change, at the individual country level however, our results still show evidence of more countries that sourced their growth from technological progress than those from improved technical efficiency. A total of 24 countries defined the PPF, 11 (45.9%) of them largely due to improved technical efficiency and 13 (54.1%) of them largely as a result of technological progress.

In general, and as shown in Table A7 of S1 Appendix, we found that out of the 44 sample countries in the study, 21 (less than 50%) recorded average TFP growth over the study period (1961–2019). Only 5 (23.8%) of them (Benin, Guinea, Sierra Leone, Tanzania and Madagascar) experienced TFP growth largely due to improvement in technical efficiency, and 16 (76.2%) of them, largely due to technological progress. The country with the highest average annual TFP growth over the study period is Zambia, with an average TFP score of 1.025 (2.5%) due to a 1.013 (1.3%) technological progress. Ghana is the only country with an average annual TFP stagnation of 1.000 coefficients resulting mainly from a recorded average annual stagnation in both technical efficiency and technological change. Comoros is identified as the least performing country with an average TFP score of 0.000 due to a decline in technology.

Further, the year 2000 happens to be the best average performing year with a 1.047 (4.7%) average annual TFP score stemming mainly from a 1.075 (7.5%) technological progress, whereas 1963 is recorded as the least average performing year for all the countries with a TFP score of 0.000, mainly as a result of technical regression, as illustrated in column 4 of Table A6 in S1 Appendix.

We also saw that out of the 59 sample years, positive average annual TFP changes were recorded in 23 (less than 50%) of those years. Three of the 59 years (1964, 1966, and 2018) recorded stagnated average annual TFP changes, 1966 and 2018 largely due to technological regression, and 1964, due to decline in efficiency. The rest of the remaining 32 years returned average annual negative TFP growth, in the region, two of them due to decline in both efficiency change and technological change, 13 (43.3%) largely due to decline in efficiency change, and 17 (56.7%) due largely to technological regression.

Our Malmquest DEA results indicate average annual decline in TFP in the SSA region during our reviewed period, largely due to technological regression. In order to give a somehow practical illustration of the average annual decline as shown in our results, outside the scope of the Malmquest DEA, we carried out further analyses on our input variables (land, labor, capital, livestock, machinery and fertilizer) to ascertain the feasibility of our results and the practical implication of dynamics in these input variables on our results. We summed the annual values of each of the input variables for all the 44 countries from 1961 to 2019, calculated the annual percentage changes in each of the variables. We further classified them on decade basis (save for 2011 to 2019, which constitutes 9 years), this gave us an understanding of the degree of percentage change that occurred in the variables for each of the decades. We calculated the average percentage change which occurred in each input variable for each decade to obtain the average decade percentage change for each of the input variables by decade. These average decade percentage changes were compared which gave us a sense of the degree of changes that occurred in the values of each input variable during each decade from 1961 through 2019.

**Fig 3:**
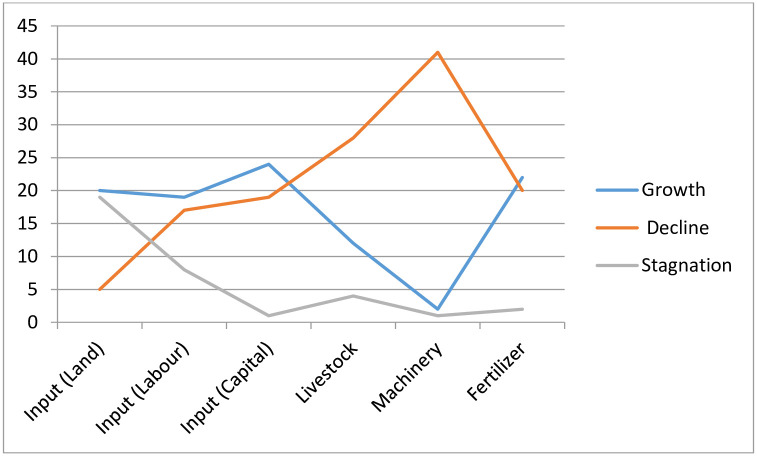
Dynamics in input variables by number of countries (1961–2019).

**Fig 4:**
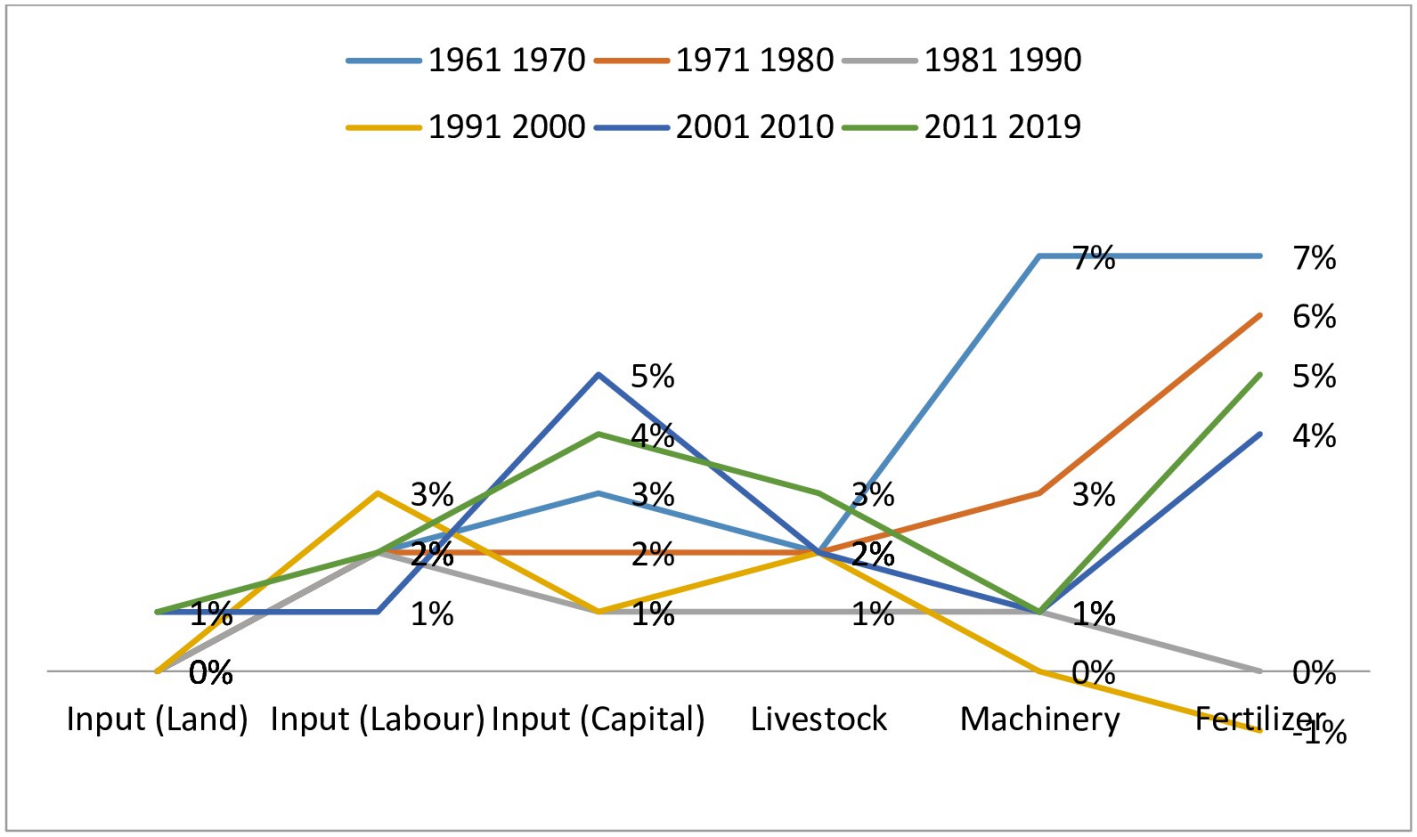
Average decade percentage change in input value (1961–2019).

The results of our analyses are presented in Figs 3 and 4. In Fig 3, our results suggest that average decade percentage growth, decline and stagnation occurred in all the input variables among the countries throughout the reviewed period. Out of the 44 countries, 20 (45.5%) recorded growth in the volume of land used, 19 (43.2%) of them, in the size of labor, 24 (54.5%) of the countries recorded growth in the volume of capital, 12 (27.3%) of them in livestock. Only two, (4.5%) of the countries (Tanzania and Somalia) recorded average decade percentage growth in the quantity of Machinery. Twenty-two, (50%) of the countries experienced average decade percentage growth in the quantity of fertilizer used during the study period. Average decade percentage decline in the volume of land used was recorded in 5, (Tanzania, Uganda, Angola, Mauritius and Zimbabwe) (11.4%) of the countries. Seventeen, (38.6%) of them recorded decline in the volume of labor, 19 (43.2%) of them in the volume of capital, 28 (63.6%) of the countries experienced average decade percentage decline in the quantity of livestock. Forty-one (93.2%) of the countries recorded average decade percentage decline the quantity of machinery and 20 (45.5%) of them in the volume of fertilizer used during the study period.

Nineteen (43%) of the countries recorded average decade percentage stagnation in the quantity of land used, eight, (18.2%) of them in labor, one country (2.3%) (Niger) recorded stagnation in the volume of capital. Four (Niger, Burundi, Namibia and South Africa) (9.1%) of the countries recorded average decade percentage stagnation in the quantity of livestock, one country (South Africa), (2.3%) in machinery and two countries (Eswatini and Liberia), (4.5%) in the volume of fertilizer used during the study period.

Fig 4 gives a detailed illustration of the average decade percentage dynamics among the six input variables. These results suggest that in general, the highest level of stagnation occurred in the volume of land used, followed by labor and livestock, machinery, fertilizer and capita, in that descending order. The highest level of growth occurred with the volume of fertilizer used and this largely occurred during the 1961–1970, 1971–1980, 2001–2010 and 2011–2019 decades, whereas the highest level of decline occurred with the use of machinery mainly during the 1991–2000, 1981–1990 and the 2011–2019 decades, suggesting that there was a general decline in the quantity or intensity of technology used in the SSA region during the study period.

This lends credence to the general results of our DEA, which indicates average annual TFP decline of 0.982 in the SSA region largely due to a 0.982 decline in technology during the reviewed period.

## 5.0 Conclusion

This work brings out significant results from our estimation of the trends and levels of agricultural productivity in the SSA region over the last five to six decades. We looked at agricultural total factor productivity change in 44 countries in the SSA region for a period of 59 years (1961–2019). Our results show an average annual TFP decline of 0.977 largely due to a 0.977 technological regression in the region. This result confirms the results of the published work of some researchers, which indicate a decline in agriculture TFP changes in the Least Developed Countries (LDCs) of the world, which includes the SSA region [9–12, 16]. This notwithstanding, we noticed intermittent positive TFP growth for some countries over a significant number of years under review. We found that 21 (less than 50%) of the 44 countries in our study recorded positive average TFP changes stemming mainly from technological progress. Out of the 59 years reviewed, we noted 23 (less than 50%) (relatively) sporadic years of positive annual average TFP changes largely due to growth in technical efficiency.

Further, our comparative analyses for 1962 and 2019 (the second and final years of our reviewed period) indicate that 21 (less than 50%) of the 44 countries defined the PPF in 1962 compared to 23 (slightly over 50%) in 2019, in each case, largely due to technological progress. Thirteen of the 21 countries which defined the PPF in 1962 successfully defined the PPF in 2019 mainly due to technical progress. Six countries (Gambia, Mauritania, Gabon, Sao Tome and Principe, Kenya, and Mauritius), all of which fell below the PPF, (with Gabon as the least performing country) in 1962, recorded TFP growth in 2019, and three of them (Gabon, Kenya, and Mauritius) largely as a result of technological progress, compared to nine of the countries, (Senegal, Cameroon, Central Africa Republic, Democratic Republic of Congo, Rwanda, Tanzania, Uganda, Somalia, and Botswana) which defined the PPF in 1962, but suffered average annual TFP decline in 2019 largely due to decline in technology. This suggests that the level of convergence and catch-up between the high and low-performing countries is relatively imbalanced. The average best performing year according to our results is the year 2000, with an average annual TFP score of 1.047 (4.7%), largely due to a 1.075 (7.5%) growth in technological change.

Generally, our DEA results for 2019 show improvement in the average annual TFP change with a score of 1.014 (1.4%), largely due to a 2.7% growth in technical efficiency, meaning that in 2019, countries became more efficient in the utilization of their existing inputs as opposed to the adoption and/or deployment of latest technologies. That said, our results show that out of the 23 countries (over 50%) which defined the production frontier in 2019, 16 sourced their growth from technological progress, which means, these countries possibly adopted multi-technological practices which aided their progress but were in the actual sense inefficient. Sao Tome and Principe, with a TFP score of 1.282 (28.2%) has the highest TFP growth for the year 2019 thus replacing Rwanda which got the highest TFP score of 1.185 (18.5%) in 1962 but fell below it in 2019. A 39% efficiency change largely accounted for the TFP growth experienced by Sao Tome and Principe; this means that Sao Tome and Principe became more efficient with its existing inputs in 2019. Côte d’Ivoire returned a decline TFP score of 0.836 (-16.4%) in 2019 which made the country the least performing country for that year. Our results indicate that such decline was as a result of a 0.836 (-16.4%) technological regression.

At the individual country level, Zambia is identified as the best average performing country, with an average annual TFP score of 1.025 (2.5%), followed by South Africa with an average annual TFP score of 1.023 (2.3%) over the study period. Comoros is the least performing country with an average annual TFP score of 0.000, largely as a result of a 0.000 negative growth in technological change.

The results of this study are a little troubling, as they reaffirm the results of some previous studies indicating average TFP decline for the SSA region. The underperformance of the region’s agriculture sector is a grave concern as the region has the fastest growing population as of 2019, compared to other regions of the world with an annual population growth rate of 2.52%. This rate of annual growth (˃2%) is projected to continue till 2040 [42]. It is thus important that the region improves on the pace of its agricultural productivity to both meet the growing demand for the products and outstrip its population growth as well. Technology has been and continues to be the main driver of agricultural productivity growth globally. Our Malmquist Index Summary of Country Means (1961–2019) as illustrated in Table A7 of S1 Appendix, suggests that 21 (less than 50%) of our sample countries recorded average annual TFP growth over the study period (1961–2019), but only 5 (23.8%) of them (Benin, Guinea, Sierra Leone, Tanzania and Madagascar) experienced TFP growth largely due to growth in technical efficiency, while 16 (76.2%) of them recorded average annual TFP growth largely due to technological progress. Further, our results indicate that 22 countries recorded average annual TFP decline between 1961 and 2019 (see Table A7 in S1 Appendix): Rwanda, as a result of stagnation in both efficiency and technological changes, Comoros as a result of a 0.000 coefficient decline in both technical efficiency and technology changes. Six (Guinea-Bissau, Gambia, Mauritania, Angola, Botswana, and Namibia), (30%) of them largely due to decline in technical efficiency, whereas 14 (70%)of them, mainly due to technological regression throughout the study period.

With our results largely pointing to a decline in total factor productivity (i.e., a decline in the ratio of agricultural output over measured inputs), the importance of policy actions and public investments to increase technical innovation and the productivity of input use in African agriculture cannot be overemphasized. As such, the effort towards agriculture output maximization to match demand should largely focus on generating and adopting improved farm technologies and management practices and adapting these technologies to be appropriate in the widely varying farming conditions found in the region. Achieving this outcome will require greater support for increased mechanization, research and development, extensive use of improved varieties that could adapt to the changing weather patterns, and process or product development.

Note that our Malmquist Index DEA results identified technological regression as a key factor for the agriculture TFP decline in the SSA region, and in line with the scope of our study, our research also established the current trends and patterns of agriculture TFP changes in the region. We believe that there are more factors influencing agricultural TFP change, therefore further research into the unconventional sources of this agricultural TFP decline is worth conducting. Such work should specifically focus on looking at the effects (if any) that agricultural policies, wars, conflicts, and political-economic systems prevalent in the SSA region have on its agricultural production capacity.

## Supporting information

S1 Appendix (DOCX)
