# Supplementary material for: Comparative performance of agricultural productivity in 44 SSA countries for a period of 59 years (1961–2019): A Malmquist productivity index approach
Source: PLoS One. 2023 Jul 24;18(7):e0284461. doi: 10.1371/journal.pone.0284461 (PMC10365310; doi:10.1371/journal.pone.0284461)
Supplement: S1 Appendix — (DOCX) [file pone.0284461.s001.docx]

**APPENDIX**

**Table A1:** Output mix in 1961 for all the 44 countries expressed in constant 2005 international dollars.

| **No.** | **Country** | **Output (Crop)** | **Output (Animal)** | **Aquaculture Output** |
| --- | --- | --- | --- | --- |
| 1 | Nigeria | 9,748,097 | 994,950 | 4,846 |
| 2 | Benin | 436,659 | 60,211 | 0 |
| 3 | Côte d'Ivoire | 1,419,586 | 174,435 | 0 |
| 4 | Ghana | 1,967,892 | 146,634 | 0 |
| 5 | Guinea | 807,148 | 95,568 | 0 |
| 6 | Guinea-Bissau | 131,012 | 25,660 | 0 |
| 7 | Liberia | 216,871 | 21,146 | 0 |
| 8 | Sierra Leone | 302,290 | 30,914 | 0 |
| 9 | Togo | 300,441 | 40,041 | 0 |
| 10 | Burkina Faso | 372,951 | 235,476 | 0 |
| 11 | Cabo Verde | 16,199 | 3,328 | 0 |
| 12 | The Gambia | 115,456 | 15,791 | 0 |
| 13 | Mali | 537,976 | 492,573 | 0 |
| 14 | Mauritania | 46,075 | 344,636 | 0 |
| 15 | Niger | 512,609 | 431,668 | 0 |
| 16 | Senegal | 969,875 | 154,690 | 0 |
| 17 | Cameroon | 1,241,863 | 243,036 | 0 |
| 18 | Central African Republic | 311,349 | 75,015 | 0 |
| 19 | Democratic Republic of Congo | 2,912,507 | 454,382 | 0 |
| 20 | Congo Republic | 158,655 | 26,088 | 0 |
| 21 | Equatorial Guinea | 70,803 | 770 | 0 |
| 22 | Gabon | 94,263 | 47,316 | 0 |
| 23 | Sao Tome and Principe | 27,051 | 1,117 | 0 |
| 24 | Chad | 479,140 | 374,202 | 0 |
| 25 | Burundi | 834,112 | 56,689 | 0 |
| 26 | Kenya | 1,127,992 | 1,042,885 | 1,929 |
| 27 | Rwanda | 672,543 | 58,633 | 0 |
| 28 | Tanzania | 1,728,476 | 645,299 | 0 |
| 29 | Uganda | 2,764,123 | 453,468 | 0 |
| 30 | Djibouti | 337 | 14,814 | 0 |
| 31 | Somalia | 159,714 | 1,056,662 | 0 |
| 32 | Angola | 996,878 | 254,104 | 0 |
| 33 | Comoros | 32,273 | 5,713 | 0 |
| 34 | Madagascar | 1,326,482 | 711,991 | 0 |
| 35 | Malawi | 577,514 | 50,444 | 0 |
| 36 | Mauritius | 237,677 | 17,104 | 0 |
| 37 | Mozambique | 1,177,888 | 164,355 | 0 |
| 38 | Zambia | 307,302 | 163,399 | 0 |
| 39 | Zimbabwe | 643,797 | 403,056 | 0 |
| 40 | Botswana | 43,642 | 154,129 | 0 |
| 41 | Eswatini | 68,676 | 79,021 | 0 |
| 42 | Lesotho | 63,040 | 75,133 | 0 |
| 43 | Namibia | 45,582 | 423,731 | 0 |
| 44 | South Africa | 3,487,174 | 3,909,373 | 0 |

**Table A2:** Output mix in 2019 for all the 44 countries expressed in constant 2005 international dollars.

| **No.** | **Country** | **Output (Crop)** | **Output (Animal)** | **Aquaculture Output** |
| --- | --- | --- | --- | --- |
| 1 | Nigeria | 52,664,781 | 5,995,347 | 650,127 |
| 2 | Benin | 3,901,743 | 366,013 | 11,405 |
| 3 | Côte d'Ivoire | 11,183,163 | 822,977 | 10,035 |
| 4 | Ghana | 12,652,906 | 921,508 | 170,942 |
| 5 | Guinea | 3,997,538 | 762,827 | 1,531 |
| 6 | Guinea-Bissau | 389,806 | 96,162 | 56 |
| 7 | Liberia | 411,846 | 108,704 | 535 |
| 8 | Sierra Leone | 1,611,130 | 172,143 | 190 |
| 9 | Togo | 1,192,167 | 262,633 | 647 |
| 10 | Burkina Faso | 3,267,874 | 1,193,155 | 961 |
| 11 | Cabo Verde | 27,322 | 20,636 | 0 |
| 12 | Gambia | 129,627 | 64,123 | 68 |
| 13 | Mali | 6,540,956 | 2,539,650 | 11,880 |
| 14 | Mauritania | 234,222 | 699,483 | 0 |
| 15 | Niger | 4,661,957 | 1,410,241 | 781 |
| 16 | Senegal | 3,120,728 | 924,699 | 1,639 |
| 17 | Cameroon | 8,892,487 | 1,075,466 | 5,218 |
| 18 | Central African Republic | 693,487 | 808,614 | 330 |
| 19 | Democratic Republic of Congo | 11,133,578 | 635,433 | 7,136 |
| 20 | Congo Republic | 462,425 | 204,224 | 1,427 |
| 21 | Equatorial Guinea | 88,919 | 1,966 | 33 |
| 22 | Gabon | 400,465 | 113,017 | 100 |
| 23 | Sao Tome and Principe | 33,247 | 4,465 | 0 |
| 24 | Chad | 2,471,739 | 3,729,676 | 197 |
| 25 | Burundi | 2,561,625 | 209,533 | 3,457 |
| 26 | Kenya | 6,854,256 | 5,978,505 | 36,051 |
| 27 | Rwanda | 2,737,619 | 527,862 | 7,932 |
| 28 | Tanzania | 12,852,518 | 4,042,074 | 37,835 |
| 29 | Uganda | 6,019,428 | 2,214,834 | 231,342 |
| 30 | Djibouti | 17,731 | 60,933 | 0 |
| 31 | Somalia | 275,384 | 1,981,240 | 0 |
| 32 | Angola | 5,121,950 | 1,135,437 | 3,907 |
| 33 | Comoros | 79,693 | 14,760 | 0 |
| 34 | Madagascar | 3,871,183 | 1,343,202 | 43,623 |
| 35 | Malawi | 7,190,496 | 1,554,204 | 20,270 |
| 36 | Mauritius | 194,102 | 117,785 | 9,883 |
| 37 | Mozambique | 3,465,610 | 833,960 | 5,919 |
| 38 | Zambia | 2,187,803 | 1,420,630 | 65,932 |
| 39 | Zimbabwe | 1,437,403 | 1,037,420 | 24,548 |
| 40 | Botswana | 72,093 | 377,420 | 56 |
| 41 | Eswatini | 349,110 | 104,916 | 223 |
| 42 | Lesotho | 59,413 | 182,677 | 11,761 |
| 43 | Namibia | 191,864 | 328,528 | 612 |
| 44 | South Africa | 10,335,201 | 11,163,950 | 16,080 |

**Table A3:** Percentage change in output quantity between 1961 and 2019.

| **No.** | **Country** | **1961** | **2019** | **Percentage change.** |
| --- | --- | --- | --- | --- |
| 1 | Nigeria | 10,747,893 | 59,306,332 | 451.8% |
| 2 | Benin | 496,870 | 4,280,560 | 761.5% |
| 3 | Côte d'Ivoire | 1,594,023 | 12,016,173 | 653.8% |
| 4 | Ghana | 2,114,526 | 13,691,231 | 547.5% |
| 5 | Guinea | 902,715 | 4,762,163 | 427.5% |
| 6 | Guinea-Bissau | 156,671 | 485,980 | 210.2% |
| 7 | Liberia | 238,017 | 521,109 | 118.9% |
| 8 | Sierra Leone | 333,204 | 1,783,462 | 435.2% |
| 9 | Togo | 340,483 | 1,457,032 | 327.9% |
| 10 | Burkina Faso | 608,427 | 4,462,063 | 633.4% |
| 11 | Cabo Verde | 19,526 | 47,994 | 145.8% |
| 12 | Gambia | 131,247 | 193,818 | 47.7% |
| 13 | Mali | 1,030,551 | 9,096,183 | 782.7% |
| 14 | Mauritania | 390,711 | 933,706 | 139% |
| 15 | Niger | 944,276 | 6,072,983 | 543.1% |
| 16 | Senegal | 1,124,563 | 4,047,329 | 259.9% |
| 17 | Cameroon | 1,484,901 | 9,973,529 | 571.7% |
| 18 | Central African Republic | 386,366 | 1,502,433 | 288.9% |
| 19 | Democratic Republic of Congo | 3,366,885 | 11,776,369 | 249.8% |
| 20 | Congo Republic | 184,742 | 668,278 | 261.7% |
| 21 | Equatorial Guinea | 71,573 | 90,917 | 27% |
| 22 | Gabon | 141,580 | 513,583 | 262.8% |
| 23 | Sao Tome and Principle | 28,169 | 37,713 | 33.9% |
| 24 | Chad | 853,343 | 6,201,610 | 626.7% |
| 25 | Burundi | 890,798 | 2,774,624 | 211.5% |
| 26 | Kenya | 2,172,805 | 12,876,128 | 492.6% |
| 27 | Rwanda | 731,175 | 3,274,067 | 347.8% |
| 28 | Tanzania | 2,373,778 | 16,933,803 | 613.4% |
| 29 | Uganda | 3,217,592 | 8,463,832 | 163% |
| 30 | Djibouti | 15,151 | 78,664 | 419.2% |
| 31 | Somalia | 1,216,376 | 2,256,624 | 85.5% |
| 32 | Angola | 1,250,983 | 6,261,681 | 400.5% |
| 33 | Comoros | 37,985 | 94,453 | 148.7% |
| 34 | Madagascar | 2,038,473 | 5,251,148 | 157.6% |
| 35 | Malawi | 627,957 | 8,763,242 | 1295.5% |
| 36 | Mauritius | 254,782 | 327,443 | 28.5% |
| 37 | Mozambique | 1,342,247 | 4,305,051 | 220.7% |
| 38 | Zambia | 470,703 | 3,694,247 | 684.8% |
| 39 | Zimbabwe | 1,046,851 | 2,502,786 | 139.1% |
| 40 | Botswana | 197,769 | 449,590 | 127.3% |
| 41 | Eswatini | 147,695 | 454,248 | 207.6% |
| 42 | Lesotho | 138,173 | 254,322 | 84.1% |
| 43 | Namibia | 469,313 | 521,082 | 11% |
| 44 | South Africa | 7,396,545 | 21,517,257 | 190.9% |

**Table A4**: Malmquist Index summary for year 2 (1962)

| **No.** | **Country** | **effch** | **techch** | **tfpch** |
| --- | --- | --- | --- | --- |
| 1 | Nigeria | 1.000 | 0.995 | 0.995 |
| 2 | Benin | 0.885 | 1.051 | 0.930 |
| 3 | Côte d'Ivoire | 1.000 | 0.774 | 0.774 |
| 4 | Ghana | 1.000 | 0.922 | 0.922 |
| 5 | Guinea | 0.941 | 1.027 | 0.966 |
| 6 | Guinea-Bissau | 0.886 | 1.089 | 0.964 |
| 7 | Liberia | 0.957 | 0.987 | 0.945 |
| 8 | Sierra Leone | 0.955 | 1.092 | 1.042 |
| 9 | Togo | 1.024 | 0.982 | 1.005 |
| 10 | Burkina Faso | 1.046 | 1.015 | 1.062 |
| 11 | Cabo Verde | 0.976 | 1.057 | 1.032 |
| 12 | Gambia | 1.000 | 0.967 | 0.967 |
| 13 | Mali | 0.946 | 1.030 | 0.974 |
| 14 | Mauritania | 1.000 | 0.951 | 0.951 |
| 15 | Niger | 0.985 | 0.946 | 0.931 |
| 16 | Senegal | 1.000 | 1.030 | 1.030 |
| 17 | Cameroon | 1.001 | 1.017 | 1.018 |
| 18 | Central African Republic | 0.956 | 1.052 | 1.006 |
| 19 | Democratic Republic of Congo | 1.000 | 1.010 | 1.010 |
| 20 | Congo Republic | 0.927 | 1.075 | 0.996 |
| 21 | Equatorial Guinea | 1.000 | 1.082 | 1.082 |
| 22 | Gabon | 1.000 | 0.414 | 0.414 |
| 23 | Sao Tome and Principe | 1.000 | 0.983 | 0.983 |
| 24 | Chad | 1.000 | 1.068 | 1.068 |
| 25 | Burundi | 1.000 | 0.992 | 0.992 |
| 26 | Kenya | 0.953 | 1.025 | 0.977 |
| 27 | Rwanda | 1.000 | 1.185 | 1.185 |
| 28 | Tanzania | 1.004 | 1.008 | 1.012 |
| 29 | Uganda | 1.000 | 1.012 | 1.012 |
| 30 | Djibouti | 1.000 | 1.001 | 1.001 |
| 31 | Somalia | 1.000 | 1.027 | 1.027 |
| 32 | Angola | 1.047 | 0.993 | 1.040 |
| 33 | Comoros | 1.000 | 0.978 | 0.978 |
| 34 | Madagascar | 0.970 | 1.055 | 1.024 |
| 35 | Malawi | 1.081 | 0.984 | 1.064 |
| 36 | Mauritius | 1.000 | 0.948 | 0.948 |
| 37 | Mozambique | 1.000 | 0.996 | 0.996 |
| 38 | Zambia | 0.968 | 1.021 | 0.989 |
| 39 | Zimbabwe | 1.037 | 1.005 | 1.042 |
| 40 | Botswana | 1.000 | 1.052 | 1.052 |
| 41 | Eswatini | 1.000 | 1.019 | 1.019 |
| 42 | Lesotho | 0.948 | 1.038 | 0.984 |
| 43 | Namibia | 1.000 | 0.857 | 0.857 |
| 44 | South Africa | 1.000 | 0.942 | 0.942 |
|  | **MEAN** | **0.988** | **0.986** | **0.974** |

**Table A5**: Malmquist Index summary for year 59 (2019)

| **No.** | **Country** | **effch** | **techch** | **tfpch** |
| --- | --- | --- | --- | --- |
| 1 | Nigeria | 1.000 | 0.985 | 0.985 |
| 2 | Benin | 1.000 | 0.955 | 0.955 |
| 3 | Côte d'Ivoire | 1.000 | 0.836 | 0.836 |
| 4 | Ghana | 1.000 | 0.908 | 0.908 |
| 5 | Guinea | 1.010 | 1.045 | 1.055 |
| 6 | Guinea-Bissau | 1.026 | 0.987 | 1.013 |
| 7 | Liberia | 0.944 | 0.962 | 0.909 |
| 8 | Sierra Leone | 1.000 | 1.048 | 1.048 |
| 9 | Togo | 1.281 | 0.956 | 1.225 |
| 10 | Burkina Faso | 1.120 | 0.972 | 1.089 |
| 11 | Cabo Verde | 1.084 | 0.930 | 1.009 |
| 12 | Gambia | 1.107 | 1.006 | 1.113 |
| 13 | Mali | 1.000 | 0.999 | 0.999 |
| 14 | Mauritania | 1.139 | 0.896 | 1.020 |
| 15 | Niger | 1.000 | 0.925 | 0.925 |
| 16 | Senegal | 1.000 | 0.952 | 0.952 |
| 17 | Cameroon | 1.000 | 0.982 | 0.982 |
| 18 | Central African Republic | 0.991 | 1.005 | 0.997 |
| 19 | Democratic Republic of Congo | 1.000 | 0.959 | 0.959 |
| 20 | Congo Republic | 1.000 | 0.945 | 0.945 |
| 21 | Equatorial Guinea | 1.000 | 1.999 | 1.999 |
| 22 | Gabon | 1.000 | 1.003 | 1.003 |
| 23 | Sao Tome and Principe | 1.390 | 0.922 | 1.282 |
| 24 | Chad | 1.000 | 1.036 | 1.036 |
| 25 | Burundi | 1.000 | 1.076 | 1.076 |
| 26 | Kenya | 1.000 | 1.045 | 1.045 |
| 27 | Rwanda | 1.000 | 0.975 | 0.975 |
| 28 | Tanzania | 1.000 | 0.994 | 0.994 |
| 29 | Uganda | 1.001 | 0.969 | 0.970 |
| 30 | Djibouti | 1.000 | 1.013 | 1.013 |
| 31 | Somalia | 1.000 | 0.994 | 0.994 |
| 32 | Angola | 1.138 | 0.897 | 1.020 |
| 33 | Comoros | 1.000 | 0.994 | 0.994 |
| 34 | Madagascar | 1.052 | 0.995 | 1.047 |
| 35 | Malawi | 1.000 | 1.020 | 1.020 |
| 36 | Mauritius | 1.000 | 1.227 | 1.227 |
| 37 | Mozambique | 0.924 | 1.033 | 0.954 |
| 38 | Zambia | 1.041 | 1.024 | 1.066 |
| 39 | Zimbabwe | 1.005 | 1.036 | 1.040 |
| 40 | Botswana | 1.005 | 0.967 | 0.973 |
| 41 | Eswatini | 1.000 | 1.022 | 1.022 |
| 42 | Lesotho | 1.000 | 1.112 | 1.112 |
| 43 | Namibia | 1.041 | 0.966 | 1.006 |
| 44 | South Africa | 1.000 | 0.975 | 0.975 |
|  | **MEAN** | **1.027** | **0.988** | **1.014** |

**Table A6**: Malmquist Index Summary of Annual Means

| Year | **effch** | **techch** | **tfpch** |
| --- | --- | --- | --- |
| 1962 | 0.988 | 0.986 | 0.974 |
| 1963 | 0.984 | 0.000 | 0.000 |
| 1964 | 0.988 | 1.012 | 1.000 |
| 1965 | 1.027 | 0.946 | 0.972 |
| 1966 | 1.002 | 0.998 | 1.000 |
| 1967 | 1.003 | 1.000 | 1.003 |
| 1968 | 0.974 | 1.000 | 0.974 |
| 1969 | 1.002 | 0.992 | 0.994 |
| 1970 | 0.938 | 1.045 | 0.981 |
| 1971 | 1.035 | 0.972 | 1.007 |
| 1972 | 0.987 | 0.990 | 0.978 |
| 1973 | 0.991 | 0.998 | 0.988 |
| 1974 | 0.998 | 1.020 | 1.018 |
| 1975 | 1.000 | 0.974 | 0.974 |
| 1976 | 1.021 | 0.963 | 0.983 |
| 1977 | 1.004 | 0.987 | 0.991 |
| 1978 | 1.027 | 0.978 | 1.004 |
| 1979 | 1.023 | 0.965 | 0.988 |
| 1980 | 1.003 | 0.986 | 0.989 |
| 1981 | 0.979 | 1.044 | 1.023 |
| 1982 | 0.985 | 1.006 | 0.990 |
| 1983 | 0.986 | 0.986 | 0.973 |
| 1984 | 1.040 | 0.968 | 1.008 |
| 1985 | 0.980 | 1.058 | 1.037 |
| 1986 | 1.032 | 0.959 | 0.989 |
| 1987 | 0.970 | 1.026 | 0.995 |
| 1988 | 1.017 | 0.992 | 1.009 |
| 1989 | 1.005 | 1.013 | 1.017 |
| 1990 | 0.991 | 0.984 | 0.975 |
| 1991 | 1.000 | 1.021 | 1.021 |
| 1992 | 0.994 | 0.981 | 0.976 |
| 1993 | 1.029 | 0.995 | 1.024 |
| 1994 | 0.996 | 1.001 | 0.997 |
| 1995 | 0.993 | 1.020 | 1.013 |
| 1996 | 1.011 | 0.994 | 1.004 |
| 1997 | 1.000 | 0.994 | 0.994 |
| 1998 | 1.005 | 1.000 | 1.005 |
| 1999 | 1.022 | 1.001 | 1.023 |
| 2000 | 0.974 | 1.075 | 1.047 |
| 2001 | 1.008 | 1.029 | 1.037 |
| 2002 | 0.978 | 1.014 | 0.992 |
| 2003 | 1.014 | 0.999 | 1.013 |
| 2004 | 0.989 | 1.006 | 0.995 |
| 2005 | 1.023 | 0.987 | 1.009 |
| 2006 | 0.998 | 0.979 | 0.977 |
| 2007 | 0.996 | 0.996 | 0.994 |
| 2008 | 1.029 | 0.989 | 1.018 |
| 2009 | 1.004 | 0.991 | 0.995 |
| 2010 | 1.011 | 1.015 | 1.026 |
| 2011 | 0.963 | 1.020 | 0.982 |
| 2012 | 1.013 | 1.007 | 1.020 |
| 2013 | 0.994 | 1.001 | 0.995 |
| 2014 | 0.997 | 0.982 | 0.979 |
| 2015 | 1.002 | 0.992 | 0.994 |
| 2016 | 0.993 | 1.001 | 0.994 |
| 2017 | 0.974 | 1.025 | 0.998 |
| 2018 | 1.024 | 0.977 | 1.000 |
| 2019 | 1.027 | 0.988 | 1.014 |
| **Mean** | **1.001** | **0.982** | **0.982** |

**Table A7**: Malmquist Index Summary of Country Means (1961 – 2019).

| **No.** | **Country** | **effch** | **techch** | **tfpch** |
| --- | --- | --- | --- | --- |
| 1 | Nigeria | 1.000 | 0.977 | 0.977 |
| 2 | Benin | 1.007 | 1.006 | 1.013 |
| 3 | Côte d'Ivoire | 1.000 | 0.989 | 0.989 |
| 4 | Ghana | 1.000 | 1.000 | 1.000 |
| 5 | Guinea | 1.002 | 1.001 | 1.004 |
| 6 | Guinea-Bissau | 0.998 | 0.999 | 0.997 |
| 7 | Liberia | 0.994 | 0.989 | 0.983 |
| 8 | Sierra Leone | 1.008 | 0.997 | 1.005 |
| 9 | Togo | 1.003 | 1.005 | 1.008 |
| 10 | Burkina Faso | 1.001 | 1.005 | 1.006 |
| 11 | Cabo Verde | 0.998 | 1.005 | 1.003 |
| 12 | Gambia | 0.990 | 0.995 | 0.985 |
| 13 | Mali | 1.001 | 1.006 | 1.007 |
| 14 | Mauritania | 0.996 | 0.999 | 0.996 |
| 15 | Niger | 1.006 | 0.991 | 0.997 |
| 16 | Senegal | 1.000 | 0.999 | 0.999 |
| 17 | Cameroon | 1.005 | 1.007 | 1.012 |
| 18 | Central African Republic | 1.004 | 1.005 | 1.010 |
| 19 | Democratic Republic of Congo | 1.000 | 1.004 | 1.004 |
| 20 | Congo Republic | 1.004 | 0.994 | 0.998 |
| 21 | Equatorial Guinea | 1.000 | 0.979 | 0.979 |
| 22 | Gabon | 1.000 | 0.986 | 0.986 |
| 23 | Sao Tome and Principe | 1.000 | 0.988 | 0.988 |
| 24 | Chad | 1.000 | 0.996 | 0.996 |
| 25 | Burundi | 1.000 | 0.978 | 0.978 |
| 26 | Kenya | 1.001 | 1.006 | 1.007 |
| 27 | Rwanda | 1.000 | 1.000 | 0.999 |
| 28 | Tanzania | 1.006 | 0.998 | 1.005 |
| 29 | Uganda | 0.999 | 0.989 | 0.988 |
| 30 | Djibouti | 1.000 | 0.988 | 0.988 |
| 31 | Somalia | 1.000 | 1.005 | 1.005 |
| 32 | Angola | 0.999 | 1.000 | 0.999 |
| 33 | Comoros | 1.000 | 0.000 | 0.000 |
| 34 | Madagascar | 1.002 | 1.001 | 1.002 |
| 35 | Malawi | 1.007 | 1.008 | 1.016 |
| 36 | Mauritius | 1.000 | 1.002 | 1.002 |
| 37 | Mozambique | 0.995 | 0.992 | 0.987 |
| 38 | Zambia | 1.012 | 1.013 | 1.025 |
| 39 | Zimbabwe | 0.999 | 1.008 | 1.007 |
| 40 | Botswana | 0.997 | 0.999 | 0.996 |
| 41 | Eswatini | 1.000 | 1.015 | 1.015 |
| 42 | Lesotho | 1.001 | 1.001 | 1.002 |
| 43 | Namibia | 0.987 | 1.003 | 0.990 |
| 44 | South Africa | 1.000 | 1.023 | 1.023 |
|  | **MEAN** | **1.000** | **0.977** | **0.977** |
